# Empirical Suitability of Scoring Systems of Observational Techniques for Repetitive Movements Based on Discomfort

**DOI:** 10.3390/healthcare11243157

**Published:** 2023-12-13

**Authors:** Dohyung Kee

**Affiliations:** Department of Industrial Engineering, Keimyung University, Daegu 42601, Republic of Korea; dhkee@kmu.ac.kr; Tel.: +82-53-580-5319

**Keywords:** joint motion, repetitive movement, discomfort, observational technique

## Abstract

This study empirically investigated the effects of repetitive movements of body parts through an experiment, and evaluated the suitability of the scoring systems of the existing observational methods for repetitive movements, based on the experimental results. Eighteen healthy college students participated in the experiment to assess discomfort, wherein joint movement, its repetition, and external load were used as independent variables. Postural loads for 16 postures used in the experiment were assessed using rapid upper limb assessment (RULA) and loading on the entire body assessment (LEBA). Three independent variables, joint motion, its repetition, and external load, as well as the interaction between motion repetition and external load, had significant effects on discomfort. Joint motion and external load significantly affected the RULA grand score, whereas all three independent variables affected the LEBA score. This finding may indicate that LEBA more accurately reflects the effect of repetitive body part movements. Additionally, the scoring systems for repetitive back motions by quick exposure check (QEC) and for repetitive wrist motions via a risk assessment and management tool for manual handling proactively II (RAMP II) may be reasonable based on the results of this study. The findings of this study can be used as reference information for better evaluation of postural loads assessed using the existing observational techniques, and as useful baseline data for the development of a new observational method to accurately assess stress caused by repetitive movements.

## 1. Introduction

Work-related musculoskeletal disorder (WMSD) is a common problem in both industrialized and developing countries [1,2]. WMSD causes various social and economic problems, such as lost productivity, medical costs, and wage compensation, as well as physical and psychological discomfort, pain, and disability [3]. In Korea, the number of WMSD cases has increased since 2018, with over 10,000 cases reported for the first time in 2021 (11,868 cases) [4]. Additionally, WMSD accounted for approximately 56–72% of the occupational diseases reported between 2010 and 2021. In 2015, the amount of compensation for WMSD under the Industrial Accident Compensation Insurance Act of Korea was approximately KRW 127 billion ($95,849,000), the third largest compensation amount after pneumoconiosis and cerebrovascular diseases [5]. Therefore, it is crucial to develop preventive measures to reduce WMSD.

Assessing exposure to WMSD is a critical step for reducing its occurrence in industry [6]. Among the various assessment methods, observational techniques have been developed frequently and used more widely in industry [7,8]. It is well known that repetitive motion or work is associated with the development of WMSDs [9,10,11,12]. However, only a few observational techniques, such as rapid upper limb assessment (RULA) [13], rapid entire body assessment (REBA) [14], quick exposure check (QEC) [15], novel ergonomic postural assessment (NERPA)) [16], risk assessment and management tool for manual handling proactively II (RAMP II) [17], loading on the entire body assessment (LEBA) [18], and modified REBA (MOREBA) [19], can assess the effects of repetitive movements.

RULA considers body posture to be repetitive only when the action is repeated more than four times per minute, without taking into account the body parts or joints involved, and it assigns an additional score of one. REBA and NERPA adopt a scoring system similar to that of RULA for repetitive movements. Based on the results of previous studies, QEC classifies the movements of the back and shoulder/arm into three classes. RAMP II divides repetitive arm movements into three categories, wrist movements and handgrip types into four categories, and MOREBA and LEBA group repetitive activities into four and five classes, respectively, based on previous studies and experimental results. Furthermore, both methods assign a score depending on the classified class without considering the body parts involved. MOREBA, QEC for the shoulder/arm, and RAMAP II for the arm only provide qualitative criteria for classifying repetitive movements. For example, RAMP II groups repetitive movements of the arm (upper and lower arms) into three qualitative classes (i.e., “constant movements mainly without pauses”, “frequent movements with some pauses”, and “varied movements, movements now and then (up to 2 times/min)”). In summary, most existing observational methods present scoring systems for repetitive movements based on subjective judgments and/or without considering the body parts used. These limitations may exist because objective data, such as experimental data, on the effects of repetitive movements for each body part are lacking, and most observational techniques have been developed simply so that they can be used more easily in industrial settings.

Therefore, this study aims to empirically investigate whether the effects of repetitive motions on discomfort differ depending on body part or joint and to assess the suitability of the scoring systems of the existing observation methods for repetitive body movements based on the experimental results. The hypotheses considered in this study are as follows:

**Hypothesis** **1:** *The effects of repetitive motions on discomfort significantly differ according to the body part or joint.*

**Hypothesis** **2:** *The scoring systems of most existing observational methods appropriately reflect the effects of repetitive body movements.*

## 2. Materials and Methods

### 2.1. Participants

Eighteen healthy college students (nine males and nine females) with no history of musculoskeletal disorders volunteered to participate in the experiment. All participants were right-handed. The whole-body discomfort of the participants was measured depending on the body part or joint motion, repetitive movement, and external load. Their demographic data are summarized in Table 1.

### 2.2. Experimental Design

Body parts or joints commonly used to perform tasks in industry, such as the wrist, elbow, shoulder, and trunk, were chosen. The joint motion of the flexion in the sagittal plane, its repetition, and external load were used as independent variables in the experiment. The level of joint motion was set to an angle often observed in industry or easily viewed by the eye (Table 2). Joint motion repetition was defined as the flexion of a joint from a neutral posture to a designated angle and back to the neutral posture. The neutral posture for all joints except the elbow was set to a joint flexion angle of 0°, whereas the neutral posture for the elbow was set to 90°.

Based on the experimental protocol and results of previous studies [20,21,22,23], two levels of joint motion repetition (6 and 12 times/min) were established. Furthermore, two levels of external load weight (0.0 kg and 2.0 kg) were used. A dumbbell (2.0 kg) was adopted as the external load. The weight of the external load was chosen based on the actual weight of hand tools used in industry (<3.0 kg) [24] and the capabilities of the participants to endure the load determined in a preliminary experiment.

The dependent variable was the whole-body discomfort measured with the Borg CR10 [25]. The participants were instructed on the scale prior to the experiment and were allowed to refer to it during the experiment.

### 2.3. Experimental Procedure

Prior to the experiment, a 10 min training session was conducted with the participants to inform them of the purpose, procedures, and potential risks involved in the experiment and to obtain informed consent. Anthropometric measurements, including stature and body weight, were taken. The experiment was conducted in a well-ventilated and well-lit laboratory at approximately 20 °C. The experimental protocol was reviewed and approved by the Institutional Review Board of Keimyung University.

**Figure 1:**
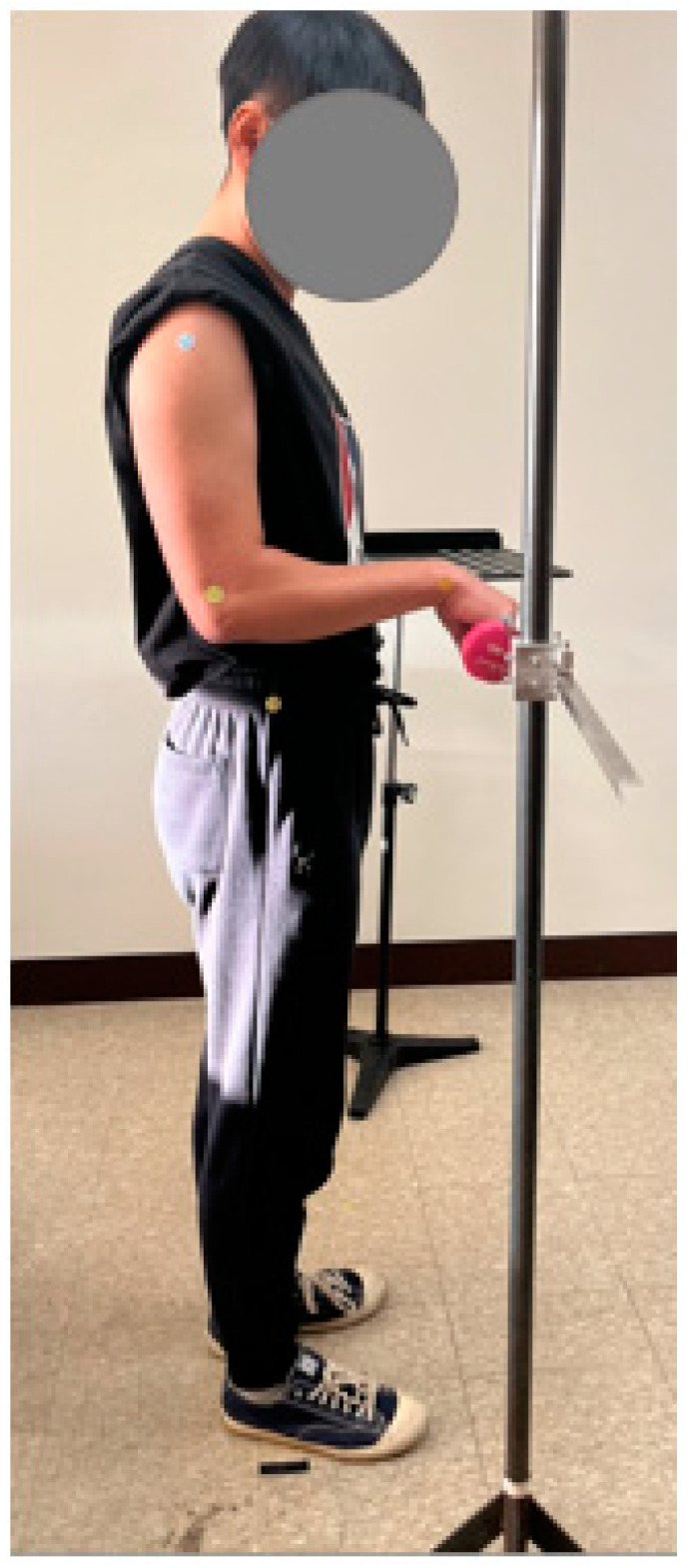
Experimental scene of wrist flexion with an external load of 2.0 kg and apparatus used in the experiment.

During the experiment, the participants were asked to wear comfortable clothing and a close-fitting short-sleeved top that exposed their arms and hands. The experimenter attached markings to the radial styloid of the wrist, lateral humeral epicondyle of the elbow, acromion of the shoulder, and iliocristale of the hip on the right side of the body as the reference points for each joint motion. The participants were required to repeat the flexion motion of each joint a given number of times for 5 min according to the experimental treatment, following the preset periodic beep sound of a computer software. In this experiment, a computer software capable of generating pre-set periodic beeps was programmed and used. In other words, the experimental task was repetitively flexing each joint from a neutral posture to a given angle according to the experimental design a given number of times for 5 min. The flexion angle of each joint was presented in Table 2. The participants were also instructed to assume neutral postures, except for the joints tested. The neutral posture is a standing position wherein the shoulder is relaxed, the right elbow is held comfortably at the side of the body and flexed by 90° (0° when performing the shoulder-relevant experimental treatments), and the right hand is in the same plane as the right forearm [26]. The left arm and hand were held vertically on the left side of the body. The external load was placed on a pedestal in front of the participants between motion repetitions. The pointer of the iron bar was used to ensure that the participants flexed the joint at an angle designated according to the experimental treatments. An experimental duration of 5 min was set according to the experimental protocol of Carey and Gallwey [20], wherein 17 wrist positions were maintained for 5 min while two levels of force (10% and 20% of the maximum voluntary contraction) were exerted at the required pace. Figure 1 shows an example of the experimental scenario for wrist flexion with a 2.0 kg external load held in the hand. The participant in Figure 1 consented to have his image published.

The participants were allowed to drop out at any time if they were unable to perform the specified experimental tasks or felt at risk of personal injury. After each experimental treatment, the participants were asked to rate their perceived whole-body discomfort using the Borg CR10. The experimental treatments were presented to each participant in a random order.

All participants were given a rest period of at least 5 min between experimental treatments. During the rest period, the participants rested in a comfortable position, such as sitting on a chair. To reduce the effect of fatigue, each participant attended two consecutive sessions on two different days for a total of 3.0–3.5 h. Each experimental session comprised eight treatments. Each session was followed by two or more warm-up tests for new postures not used in the experimental conditions.

### 2.4. Data Analysis

Firstly, an analysis of variance (ANOVA) was performed to examine the statistical significances of the independent variables as well as the gender and body mass index (BMI) of the participants on the whole-body discomfort as measured by the Borg CR10. The BMIs were classified into six categories according to the ranges set out by the Korean Society for the Study of Obesity: <18.5—Underweight; 18.5~22.9—Normal range; 23.0~24.9—Overweight (pre-obese); 25.0~29.9—Obese (Class I); 30~34.9—Obese (Class II); ≥35.0—Obese (Class III) [27]. The effects of the independent variables were analyzed using the Student–Newman–Keuls (SNK) test and plotting the discomfort with respect to independent variables. Secondly, the author evaluated postural loads for the 16 experimental treatments studied during the experiment using RULA and LEBA posture classification schemes to check whether the two techniques adequately reflected the effect of repetitive motion. This step resulted in four postural load scores for each experimental treatment: a grand score and an action level for RULA, a LEBA score, and an action category for LEBA. ANOVA and the chi-square test were used to analyze the effects of independent variables on the continuous data of RULA grand scores and LEBA scores, and the categorical data of RULA action levels and LEBA action categories, respectively. All statistical analyses were performed using SAS (SAS Inc., Cary, NC, USA) and Microsoft Excel (Microsoft Corp., Redmond, WA, USA).

## 3. Results

### 3.1. Effects of Independent Variables on Discomfort

ANOVA was performed to analyze the significances of the independent variables as well as the gender and BMI of the participants on discomfort, and the results are shown in Table 3. All independent variables, i.e., the body part or joint motion, motion repetition, and external load, had significant effects on discomfort at α = 0.01. Although the interaction effect between joint motion repetition and external load was significant at α = 0.01, the effects between joint motion and its repetition, and between joint motion and external load, were not significant (*p* > 0.10). The gender and BMI of the participants significantly affected discomfort (*p* < 0.01).

**Figure 2:**
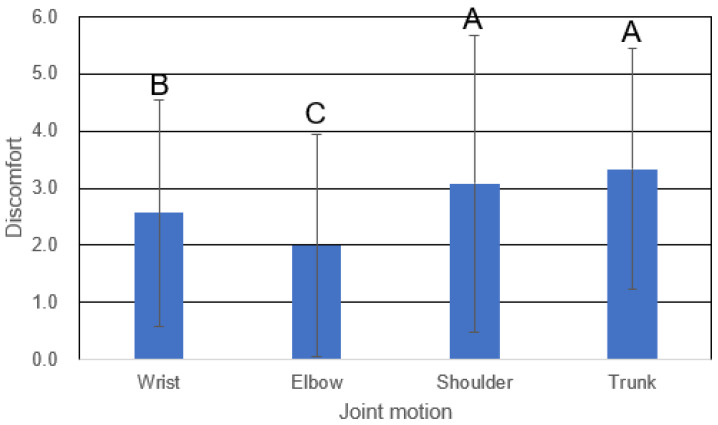
Discomfort grouped by joint motion (letters A, B, and C represent the discomfort groupings according to the SNK test).

**Figure 3:**
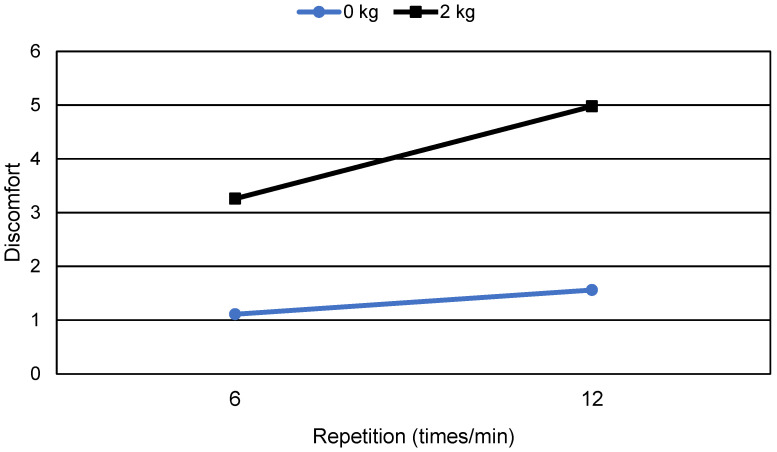
Interaction effect of discomfort between joint motion repetition and external load.

SNK tests were performed to classify the effects of the significant independent variables on discomfort (α = 0.05). The effects of joint motion were grouped into three classes based on discomfort: elbow, wrist and shoulder, and trunk (Figure 2). The effect of the elbow joint motion on discomfort was the lowest, followed by those of the wrist, shoulder, and trunk motion. The effects of joint motion repetition and external load were classified into two groups based on their experimental levels. Figure 3 shows the interaction effect between joint motion repetition and external load, indicating that the effect of joint motion repetition increases as external load increases. The *t*-test results demonstrated that the effect of joint motion repetition on discomfort was not significant in the wrist (*p* > 0.10), whereas the effects for the other joint motions were significant at α = 0.05.

### 3.2. Effects of Independent Variables on RULA Grand Score and LEBA Score

Among the observation techniques reflecting the risk factors of repetitive joint motion, RULA and LEBA provided the RULA grand and LEBA scores, combining the scores of the risk factors considered in them. ANOVA was performed to examine the significances of the independent variables on the RULA grand and LEBA scores (Table 3). The RULA grand and LEBA scores were assumed to be interval scales. The effects of joint motion and external load had statistically significant effects on the RULA grand score at α = 0.05. The *p*-value for joint motion repetition was 1.0 because the RULA scoring system assigned the same score of one, regardless of the two levels of joint motion repetition chosen in the experiment (6 and 12 times/min). Joint motion and its repetition had a significant effect on the LEBA score at α = 0.05, and external load did at α = 0.01.

A chi-square test was used to examine the significances of the independent variables on the RULA action level and LEBA action category, which are categorical data. Although the effects of joint motion and repetition were not significant on the RULA action level and LEBA action category (*p* > 0.10), the effect of external load was significant (*p* < 0.01).

### 3.3. Scoring Systems for Repetitive Movements

The effect of joint motion repetition on discomfort was significantly different depending on the levels (6 and 12 times/min) in the ANOVA results (Table 3). However, RULA, REBA, and NERPA gave a score of one for repetitive movements of >4 times/min. Thus, no significant effect of joint motion repetition on the RULA grand score was evident, whereas the effect on the LEBA score was significant (Table 3). LEBA classifies repetitive joint motion into five categories and divides the two levels of joint motion repetition used in the experiment into different groups (i.e., 6–10 and 11–15 times/min, respectively).

QEC classifies repetitive back movements into three classes (i.e., around 3 times/min or less, around 8 times/min, and around 12 times/min or more), and RAMP II divides repetitive wrist motion into four classes (i.e., more than 20 times/min, 11–20 times/min, 6–10 times/min, and up to 5 times/min). The two methods classified the two levels of motion repetition used in this study into two different classes (around 8 times/min and around 12 times/min or more in QEC; 6–10 times/min and 11–20 times/min in RAMP II). These classifications can be considered reasonable, based on the significances of the two levels of motion repetition included in this study.

MOREBA provides five classes for repetitive activities, irrespective of the joint movements involved, and QEC and RAMP II use three classes for arm movements. The adequacy of these classification schemes could not be examined based on the results of this study, considering that the above three methods qualitatively classified joint motion repetitions without the quantitative classification criteria. For example, the five classes of MOREBA for repetitive activity are “never”, “little”, “sometimes”, “much”, and “very much”.

## 4. Discussion and Conclusions

This study empirically investigated the effects of the body part or joint movement, its repetition, and external load on discomfort, the RULA grand score, and the LEBA score. The results showed that three independent variables—joint motion, repetition, and external load—as well as an interaction between joint motion repetition and external load had significant effects on discomfort. We can conclude that an observational technique should be equipped with a scoring system that reflects the effect of repetitive movement for precisely assessing postural loads. Additionally, the joint motion and external load had significant effects on the RULA grand score, whereas all three independent variables did on the LEBA score. This finding may suggest that LEBA more accurately assesses postural loads induced by repetitive body movements compared to RULA as well as REBA and NERPA, which are similar to RULA. The results of this study can be used as reference information for evaluating postural loads assessed by using existing observational methods, such as RULA, REBA, NERPA, and LEBA, and useful basic data to develop a new observational technique to more adequately reflect the effect of repetitive movement.

The insignificance of the effect of motion repetition on the RULA action level is an inherent problem of RULA, which reflects the effect of repetitive movements depending on whether a movement is repeated 4 times/min or more. However, the insignificance of the LEBA action category with five classes of movement repetition can be surmised because the levels of movement repetition used in the experiment (6 and 12 times/min) were not sufficiently high to require corrective action for the experimental postures.

The author originally expected that the effect of movement repetition would vary depending on the body part or joint motion (i.e., the interaction between joint movement and repetition would be significant). To confirm this expectation and quantify the effect, joint movement and repetition were included as independent variables in the experiment. However, the experimental results showed that the interaction between these two factors was not statistically significant. That is, in the experiment conducted in this study, the effect of movement repetition was not statistically significantly different depending on the joint motion. This result may be due to the light external load of 2.0 kg or less, which was supported by the fact that the interaction between joint movement and external load was not significant and that the level of movement repetition adopted in this study was not lower than those in previous studies. Kilbom estimated the risk of shoulder motion > 2.5 times/min, upper arm and elbow motion > 10 times/min, and forearm and wrist motion > 10 times/min as high [23]. The results of this study, which showed that the effect of repetitive motion on the wrist was not significant but those for the elbow, shoulder, and trunk were significant, are in general agreement with those obtained by Kilbom [23]. Carey and Gallwey [20], following the work of Yen and Radwin [22], examined two levels of wrist motion pace, 10 and 20 exertions/min, which showed that the effect of wrist repetitive motion was not significant (*p* > 0.10).

The above results may demonstrate the validity of various observational techniques, such as RULA, REBA, NERPA, LEBA, and MOREBA, which evaluate the stress of repetitive motion without considering joint motion, especially when the external load or exertion is light, as in this study. However, based on the findings of this study, the scoring systems for motion repetition by RULA, REBA, and NERPA with only two classes of motion repetition (i.e., <4 times/min and ≥4 times/min) may underestimate the stress induced by repetitive motion, whereas the repetitive movement rating system of LEBA (five quantitative classes), repetitive back movement rating system of QEC (three quantitative classes), and repetitive wrist motion rating system of RAMP II (four quantitative classes) may better assess postural stress.

Although the interaction between motion repetition and external load had a significant effect on discomfort, no observational technique differentially reflects the effect of motion repetition depending on the magnitude of the external load. Therefore, a new observational technique that considers this factor may be required to accurately assess postural loads in industry.

It was difficult for the participants to bear an external load heavier than the 2.0 kg used in this study, as found in the preliminary experiment of this study. If the external load is heavier, the effect of external load may vary depending on the joint motion unlike the result of this study, as discussed above. Therefore, it is recommended that the effect of motion repetition for heavy external load or exertion be investigated based on epidemiological studies rather than empirical studies, such as experiments as in this study.

Among the various observational techniques, RULA and LEBA were used to assess the postural loads of the 16 experimental treatments performed in this study. These methods were chosen because RULA has been most commonly used in the United States, Canada, and the United Kingdom [28], and LEBA is considered to be better than RULA for assessing postural loads and predicting their association with WMSDs [29].

This study investigated the effects of joint motion, its repetition, and external load. It also looked at the suitability of the scoring systems of the existing observational methods based on subjective discomfort rather than objective measures such as biomechanical stress and epidemiological data. However, this approach is supported by the findings of previous studies that reported that (1) discomfort is a valid measure of postural load [30]; (2) minimizing discomfort can help to reduce the risk of musculoskeletal disorders [31]; (3) discomfort can be considered as an independent evaluation criterion for static postures [31]; and (4) discomfort provides a simple and versatile means for assessing postural stress [32].

Although this study included multiple joints such as the wrist, elbow, shoulder, and trunk, which are commonly used in industrial work, only two levels of joint motion repetition and external load were adopted to reduce the experimental size. Further, only the joint motion of the flexion in the sagittal plane was used in the experiment. Additionally, this study was conducted for a small population of 18 college students. A follow-up study with different joint motions, such as extension, rotation, and adduction/abduction, and more levels of motion repetition and external load for a more diverse and larger population of participants is needed to understand the effect of motion repetition more fully according to each body part. Furthermore, the development of a new observational technique is recommended to more effectively evaluate musculoskeletal risk factors, which include work-related factors of vibration, contact stress, extreme temperature, and length of service; individual differences in physical ability, health conditions, and work habits; and psychosocial, environmental, and organizational factors.

## Figures and Tables

**Table 1:** Demographic data of study participants.

Variables	Total (N = 18) Mean ± SD *	Males (N = 9) Mean ± SD	Females (N = 9) Mean ± SD
Age (years)	21.1 ± 1.43	21.2 ± 1.86	20.9 ± 0.93
Stature (cm)	168.5 ± 10.23	177.6 ± 3.24	159.5 ± 5.33
Body weight (kg)	−65.4 ± 15.82	79.7 ± 8.05	51.1 ± 2.85

*: standard deviation.

**Table 2:** Independent variables and their levels.

Body Part	Flexion Angle (°)	Repetition (times/min)	External Load (kg)
Wrist	30	6, 12	0, 2
Elbow	135		
Shoulder	60		
Trunk	45		

**Table 3:** Results of ANOVA.

Dependent Variable	Source	DF	Mean Square	F Value	Pr > F
Discomfort	Joint motion	3	20.83	11.02	<0.01
	Repetition	1	82.70	43.78	<0.01
	External load	1	481.26	254.74	<0.01
	Gender	1	102.56	54.29	<0.01
	BMI	3	16.39	8.67	<0.01
	Joint motion * Repetition	3	1.42	0.76	>0.10
	Joint motion * External load	3	0.70	0.37	>0.10
	Repetition * External load	1	23.12	12.24	<0.01
RULA grand score	Joint motion	3	0.92	12.22	<0.01
	Repetition	1	0.00	0.00	1.00
	External load	1	20.25	270.00	<0.01
LEBA score	Joint motion	3	12.77	6.18	<0.05
	Repetition	1	17.64	8.53	<0.05
	External load	1	264.88	128.13	<0.01

## Data Availability

The datasets used and/or analyzed during the current study are available from the corresponding author on reasonable request.
